# Supplementary material for: Paranoia and conspiracy: group cohesion increases harmful intent attribution in the Trust Game
Source: PeerJ. 2019 Aug 16;7:e7403. doi: 10.7717/peerj.7403 (PMC6699476; doi:10.7717/peerj.7403)
Supplement: Supplemental Information 1 — Statistical models, top model sets, experiment materials and instructions [file peerj-07-7403-s001.docx]

**Title:**

**Paranoia and conspiracy: group cohesion increases harmful intent attribution in the Trust Game**

**Anna Greenburgh^a^, Vaughan Bell^b,c^ & Nichola Raihani^a^**

a. Department of Experimental Psychology, University College London, 26 Bedford Way, London WC1H 0AP, England

b. Division of Psychiatry, University College London, Maple House, 149 Tottenham Court Road, London W1T 7BN.

c. South London and Maudsley NHS Foundation Trust

**Corresponding author:**

[**a.greenburgh@ucl.ac.uk**](mailto:a.greenburgh@ucl.ac.uk)

**+44 (0) 7546660060**

**Contents**

Statistical Methods (p. 2)

Table S1: Top model sets for cumulative link model for harmful intent attributions (p. 3)

Table S2: Top model set for cumulative link model for self-interest attribution (p. 4)

Table S3: Subject responses to the question of how confident they were that they were playing against a real person (p. 5)

Table S4: Subject responses to the question of how whether they had participated in tasks similar to this previously (p. 5)

Table S5: Subject responses to the manipulation check (p. 5)

The Green et al. Paranoid Thoughts Scale (p. 6-8)

Game Instructions for Proposers on Amazon Mechanical Turk (p. 9)

Game Instructions for Responders at UCL (p16)

References (p22)

#

# Statistical Method

To compare the relative effect sizes and confidence intervals associated with input variables in the models, we used multi-model selection with model averaging (described in detail in Burnham & Anderson 2002 and Grueber et al. 2011). This approach involves specifying a priori a candidate set of possible models and then comparing across all these models to see which model, or subset of models, is most consistent with the data. Models are compared using an AIC value (AICc, corrected for small sample sizes), which includes a penalty for including additional terms in the model. Lower AIC values indicate that the model is more consistent with the observed data. For each analysis, we first specified a global model containing all fixed effects and specified interactions. All possible models deriving from this global model are compared, resulting in a top model set containing all models that are within 2 AICc units of the ‘best’ model (that with the lowest AICc value). Parameter estimates are obtained by averaging across this top model set, thereby incorporating the uncertainty over the true parameter estimate when many models have similar levels of support. We report full model estimates and confidence intervals, which are conservative.

| **Rank** | **Parameters** | **df** | **AICc** | ***w^i^*** |  |
| --- | --- | --- | --- | --- | --- |
| **1** | **Cohesion + Fairness + Gender + Comprehension + Trust + Paranoia** | **10** | **2588.37** | **0.68** |  |
| 2 | | Cohesion + Fairness + Gender + Comprehension + Trust + Paranoia + Cohesion:Paranoia | 11 | 2589.83 | 0.32 |
|  | |  |  |  |  |

**Table S1.** Top model set for the cumulative link model investigating the attribution of harmful intent to two responders in the trust game. Models are ranked in decreasing order of the support, based on relative AIC values. Akaike weights (*w^i^*) give the probability that each model is the true best model.

| **Rank** | **Parameters** | | **df** | **AICc** | ***w^i^*** |  |
| --- | --- | --- | --- | --- | --- | --- |
| **1** | **Fairness** | **5** | | **2507.13** | **0.17** |  |
| 2 | | Fairness + Gender | 6 | | 2507.25 | 0.16 |
| 3 | | Fairness + Trust decision | 6 | | 2507.93 | 0.11 |
| 4 | | Fairness + Gender + Trust decision | 7 | | 2508.14 | 0.10 |
| 5 | | Fairness + Gender + Comprehension | 7 | | 2508.15 | 0.10 |
| 6 | | Fairness + Comprehension | 6 | | 2508.18 | 0.10 |

**Table S2.** Top 6 model set for the cumulative link model investigating the attribution of self-interest to two responders in the trust game. Models are ranked in decreasing order of the support, based on relative AIC values. Akaike weights (*w^i^*) give the probability that each model is the true best model.

| **1**  **(not at all)** | **2** | **3** | **4** | **5**  **(extremely)** |
| --- | --- | --- | --- | --- |
| 138  12% | 121  11% | 199  17% | 330  29% | 354  31% |

**Table S3.** Subject responses to the question of how confident they were that they were playing against a real person.

| **1**  **(nothing like this)** | **2** | **3** | **4** | **5**  **(exactly like this)** |
| --- | --- | --- | --- | --- |
| 141  12% | 153  13% | 379  33% | 395  35% | 74  6% |

**Table S4.** Subject responses to the question of whether they had participated in tasks similar to this previously.

|  | | **Perceived cohesiveness** | | |
| --- | --- | --- | --- | --- |
| **Condition** |  | **Cohesive** | **Non-cohesive** | **Unsure** |
|  | **Cohesive** | 486  83% | 26  4% | 75  13% |
|  | **Non-cohesive** | 15  3% | 504  89% | 45  8% |

**Table S5.** Subject responses to the manipulation check for cohesive. Percentages are calculated per condition.

**The Green et al. (2008) Paranoid Thoughts Scale**

To begin, **please enter your Amazon Mechanical Turk WorkerID** here:

(Please see below for where you can find your Worker ID.)

Your Worker ID starts with the letter A and has 12-14  letters or numbers. It is NOT your email address. If we do not have your correct Worker ID we will not be able to pay you.

Your WorkerID can be found on your dashboard page.

You are about to take part in an academic study which is run by the Raihani Lab, based at University College London. This project has been approved by the UCL Ethics Board project 3720/001.

By continuing, you are consenting to allow the Raihani Lab to use your responses in the study for academic purposes.

The purpose of this study is to understand people's behaviour. All data are anonymous (your name will not appear in any publication related to this study and will not be shared with any other parties). By completing this HIT you will be granted a qualification to participate in a subsequent HIT run by Raihani Lab in the future. You will be notified when the next HIT becomes available.

Please tick 'I agree' if you agree to these conditions. If you do not wish to participate, or if you change your mind during the course of the study, please close this window.

- I agree (1)

Please read each of the following statements carefully. They refer to thoughts and feelings you may have had about others **over the last month**. Think about last month and indicate the extent of these feelings from **1 (Not at all) to 5 (Totally).**

**Please complete both Part A and Part B.**

(N.B. Please do not rate items according to any experiences you may have had under the influence of drugs.)

**Part A:**

|  | Not at all (1) | 2 | 3 | 4 | Totally (5) |
| --- | --- | --- | --- | --- | --- |
| 1. I spent time thinking about friends gossiping about me. |  |  |  |  |  |
| 2. I often heard people referring to me. |  |  |  |  |  |
| 3. I have been upset by friends and colleagues judging me critically. |  |  |  |  |  |
| 4. People definitely laughed at me behind my back. |  |  |  |  |  |
| 5. I have been thinking a lot about people avoiding me. |  |  |  |  |  |
| 6. People have been dropping hints for me. |  |  |  |  |  |
| 7. I believed that certain people were not what they seemed. |  |  |  |  |  |
| 8. People talking about me behind my back upset me. |  |  |  |  |  |
| 9. I was convinced that people were singling me out. |  |  |  |  |  |
| 10. I was certain that people have followed me. |  |  |  |  |  |
| 11. Certain people were hostile towards me personally. |  |  |  |  |  |
| 12. People have been checking up on me. |  |  |  |  |  |
| 13. I was stressed out by people watching me. |  |  |  |  |  |
| 14. I was frustrated by people laughing at me. |  |  |  |  |  |
| 15. I was worried by people's undue interest in me. |  |  |  |  |  |
| 16. It was hard to stop thinking about people talking about me behind my back. |  |  |  |  |  |

**Part B:**

|  | Not at all (1) | 2 | 3 | 4 | Totally (5) |
| --- | --- | --- | --- | --- | --- |
| 1. Certain individuals have had it in for me. |  |  |  |  |  |
| 2. I have definitely been persecuted. |  |  |  |  |  |
| 3. People have intended me harm. |  |  |  |  |  |
| 4. People wanted me to feel threatened, so they stared at me. |  |  |  |  |  |
| 5. I was certain people did things in order to annoy me. |  |  |  |  |  |
| 6. I was convinced there was a conspiracy against me. |  |  |  |  |  |
| 7. I was sure someone wanted to hurt me. |  |  |  |  |  |
| 8. I was distressed by people wanting to harm me in some way. |  |  |  |  |  |
| 9.I was preoccupied with thoughts of people trying to upset me deliberately. |  |  |  |  |  |
| 10. I couldn't stop thinking about people wanting to confuse me. |  |  |  |  |  |
| 11. I was distressed by being persecuted. |  |  |  |  |  |
| 12. I was annoyed because others wanted to deliberately upset me. |  |  |  |  |  |
| 13. The thought that people were persecuting me played on my mind. |  |  |  |  |  |
| 14. It was difficult to stop thinking about people wanting to make me feel bad. |  |  |  |  |  |
| 15. People have been hostile towards me on purpose. |  |  |  |  |  |
| 16. I was angry that someone wanted to hurt me. |  |  |  |  |  |

**Game instructions for Proposers on Amazon Mechanical Turk**

**Where instructions and/or information was the same across both tasks, we do not duplicate instructions.**

***General Consent Screen***

You are about to take part in an academic study, which is run by the Raihani Lab, based at University College London. This project has been approved by the UCL Ethics Board project 3720/001.

**Please note that the Raihani Lab does not use deception. All participants are real.**

By continuing, you are consenting to allow the Raihani Lab to use your responses in the study for academic purposes.   The purpose of this study is to understand people's behaviour. All data are anonymous (your name will not appear in any publication related to this study and will not be shared with any other parties).

Please tick 'I agree' if you agree to these conditions. If you do not wish to participate, or if you change your mind during the course of the study, please close this window.

- I agree

***Starting information***

You are **Player A.**

You will interact with **Player B & Player C.**
You will play a game where your decisions and those of **Players B** **& C** will determine your earnings.

**Player B** **&** **Player C** are **not** MTurk workers - they are participants at University College London.
The decisions made by Player B & Player C in this task have already been recorded.
Your worker ID will not be revealed to Player B or Player C.

***Cohesive condition game description***

In this game you start with $0.50

You can choose whether to send $0.05 or $0.25 to **Player B** **&** **C's team.**

You keep whatever you do not send

The amount you send to Player B & C's team will be **multiplied by 4.**

**If you send $0.05, it will be increased to $0.20**

**If you send $0.25, it will be increased to $1.00**

Players B & C can jointly decide whether to send half back to you, or to keep all the money for themselves.

**If you send $0.25:**

and Players B & C **send half back** to you, you will get **$0.75**

and Players B & C **keep all the money**, you will get **$0.25**

**If you send $0.05:**

and Players B & C **send half back** to you, you will get **$0.55**

and Players B & C **keep all the money**, you will get **$0.45**

**Players B & C discussed and agreed on how much of the money they wanted to send back to you.**

**The decision you will see is therefore the decision that was agreed upon by both players.**

***Non-cohesive condition game description***

In this game you start with $0.50

You can choose whether to send $0.05 or $0.25 to **Player B** **&** **Player C**

You keep whatever you do not send

Any amount you send to Player B & Player C will be **multiplied by 4.**

**If you send $0.05, it will be increased to $0.20**

**If you send $0.25, it will be increased to $1.00**

Players B & C independently decide whether to send half back to you, or to keep all the money for themselves.

**If you send $0.25:**

and Players B and C **send half back** to you, you will get **$0.75**

and Players B and C **keep all the money**, you will get **$0.25**

**If you send $0.05:**

and Players B and C **send half back** to you, you will get **$0.55**

and Players B and C **keep all the money**, you will get **$0.45**​

**Players B & Player C could not communicate in this task.**

**The decision you will see was made by both players independently - they did not make this decision together.**

***Comprehension questions***

First, please answer three questions to confirm you have understood the game.

Q1.  If you send $0.25 to Players B & C, how much will this be increased to?

- $0.50
- $1.00
- It won’t be increased at all

Q2. Which decision will give Players B & C the highest earnings?

- Return nothing back to you
- Return half the amount they receive back to you

Q3. Do Players B & C jointly decide how they should divide the money?

- Yes
- No

***Decision***

Of your $0.50 bonus, do you want to send $0.25 or $0.50 to Players B & C

- Send $0.25 to Players B & C, which will be increased to $1.00
- Send $0.05 to Players B & C, which will be increased to $0.20

***Feedback***

*Fair cohesive responders*

You played against Players B & C who decided together to send back half of the money to you

*Unfair cohesive responders*

You played against Players B & C who decided together to send back none of the money to you

*Fair non-cohesive responders*

You played against Players B & C who decided separately to send back half of the money to you

*Unfair non-cohesive responders*

You played against Players B & C who decided separately to send back none of the money to you

***Intention Attribution***

Please use the slider below to indicate the extent to which you believe Player B & C’s decisions were driven by their desire to earn money in the game.

Not at all Completely


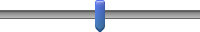


Please use the slider below to indicate to what extent Player B & C’s decisions were driven **by their desire to reduce your bonus** in the game.

Not at all Completely


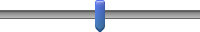


***Manipulation check***

To what extent do you think Players B & C made their decision as a team?

- Players B & C decided as a team how much of the bonus to send back to me
- I’m not sure
- Players B & C made their decision separately

*Demographic and other questions*

Thanks - it's nearly the end! Before you go, it would be very helpful if you could answer some additional questions to help with our research.

Unlike some other research labs, Raihani Lab **does not** use deception on MTurk. All participants are real. Nevertheless, for our own purposes, it is helpful to know to what extent you believed that the other player really existed.

|  | 1 - Very skeptical that other player was real (1) | 2 (2) | 3 (3) | 4 (4) | 5 -Very confident that other player was real (5) |
| --- | --- | --- | --- | --- | --- |
| Please choose one (1) |  |  |  |  |  |

To what extent have you participated in HITs similar to this before?

|  | 1 - Nothing like this scenario (1) | 2 (2) | 3 (3) | 4 (4) | 5 - Exactly like this scenario (5) |
| --- | --- | --- | --- | --- | --- |
| Please choose one (1) |  |  |  |  |  |

What is your age?

________________________________________________________________

What is your gender

- Male
- Female
- Prefer not to say

**Game instructions for responders at UCL**

***Cohesive responder instructions***

In this project you have been assigned the role of Player C. **Your identity will be kept anonymous from all other players.**

You will be paired with a **teammate,** Player B, also a participant from UCL, who is taking part at the same time, but in a different room so you remain anonymous. You will later be required to talk to each other online. **It is vital that you do not reveal your identity to Player B at anytime.**  You will both be playing against another participant, Player A, whose decisions will be collected at a later date.

In the game you play, you and Player B will make your decisions as a team. These decisions, along with those of Player A, will determine your earnings.

In this game, Player A will be given an endowment of $0.50. They have two choices as to what to do with this money. They can either…

- Send you and Player B $0.05 and keep $0.45 for themself
- Send you and Player B $0.25 and keep $0.25 for themself

Any amount they send to you will be quadrupled.

You and your teammate, Player B, decide **together** what to do with this quadrupled sum that Player A sends you. You can either…

- Keep **all** of it and return $0 to Player A
- Keep **half** of it and return half of it to Player A

Note whatever you keep will be split equally between you and Player B.

The game structure and your payoffs are depicted in Figure 1.

**Your task:** After reading and understanding this please tell the experimenter. You will first be asked to talk online with Player B and come to a mutual decision regarding, for every possible decision from Player A, what your response will be.

Please give your answer on the response form.

**Payment:** You will be paired at random with multiple Player As, and the decision you give will be applied to every one of these situations. Therefore you will earn more than the amount noted here.

For example, if you are paired with 100 Player As who decide to send you $0.25, and you have indicated that if they do this, you want to keep the entire sum, then your final payoff will be $25. You will be paid this amount in **amazon vouchers.** Please give us your email address so we can send these to you

***Non-cohesive responder instructions***

In this project you have been assigned the role of Player C. **Your identity will be kept anonymous from all other players.**

You will be paired with Player B, also a participant from UCL.

You will both be playing against an online participant, Player A, whose decisions will be collected at a later date.

In the game you play, your decisions, along with Player A and Bs’ decisions, will determine your earnings.

In this game, Player A will be given an endowment of $0.50. They have two choices as to what to do with this money. They can either…

- Send you and Player B $0.05 and keep $0.45 for themself
- Send you and Player B $0.25 and keep $0.25 for themself

Any amount they send to you will be quadrupled.

You and Player B each decide separately what to do with this quadrupled sum that Player A sends you. You can either…

- Keep **all** of it and return $0 to Player A
- Keep **half** of it and return half of it to Player A

Note whatever you keep will be split equally between you and Player B. You will be matched with a player B who makes the same decision as you.

The game structure and your payoffs are depicted in Figure 1.

**Your task:** considering this, please indicate for every possible decision from Player A, what your response would be. Please give your answer on the response form.

***Figures shown to responders***

- 1. ***Cohesive***

******

- 1. ***Non-cohesive***

***Response***

If Player A sends $0.25, it will be quadrupled to $1.00. Out of this $1.00, I want to return Player A….

- Half ($0.50)
- None

If Player A sends $0.05, it will be quadrupled to $0.20. Out of this $0.20, I want to return Player A…

- Half ($0.10)
- None

***References***

Green CEL, Freeman D, Kuipers E, Bebbington P, Fowler D, Dunn G, et al. Measuring ideas of persecution and social reference: the Green et al. Paranoid Thought Scales (GPTS). Psychological Medicine. 2007;38(01).
